# Investigating the disturbance in cortical glutamate and GABA function in psychosis and its origins and consequences

**DOI:** 10.1038/s41380-025-03337-x

**Published:** 2025-11-19

**Authors:** Bill Deakin, Elizabeth Liddle, Mohanbabu Rathnaiah, Catherine C. Gregory, Mohammad Z. Katshu, Gemma Williams, Silke Conen, Richard Smallman, Loes C. Koelewijn, Adriana Anton, Jyothika Kumar, Lauren E. Gascoyne, Chen Chen, Naghmeh Nikkheslat, John Evans, Bernard Lanz, James Walters, Peter S. Talbot, Lena Palaniyappan, Krish D. Singh, Peter Morris, Stephen R. Williams, Peter F. Liddle

**Affiliations:** 1 Division of Psychology and Mental Health, University of Manchester, Manchester Academic Health Sciences Centre, M13 9PT Manchester, UK; 2 Institute of Mental Health, Division of Mental Health and Clinical Neuroscience, University of Nottingham, Nottingham, NG7 2TU UK; 3 Nottinghamshire Healthcare NHS Foundation Trust, Nottingham, NG3 6AA UK; 4 CUBRIC, School of Psychology, College of Biomedical and Life Sciences, Cardiff University, Cardiff, CF24 4HQ UK; 5 Division of Medical Education, University of Manchester, M19 3PT Manchester, UK; 6 Sir Peter Mansfield Imaging Centre, University of Nottingham, Nottingham, NG7 2RD UK; 7 Stress, Psychiatry and Immunology Lab & Perinatal Psychiatry, The Maurice Wohl Clinical Neuroscience Institute, King’s College London UK, London, UK; 8 CIBM Center for Biomedical Imaging, Lausanne, Switzerland; 9 Animal Imaging and Technology, EPFL, Lausanne, Switzerland; 10 MRC Centre for Neuropsychiatric Genetics and Genomics, Cardiff University, CF24 4HQ Cardiff, United Kingdom; 11 Robarts Research Institute, Western University, London, ON Canada; 12 Department of Medical Biophysics, Western University, London, ON Canada; 13 Department of Psychiatry, Western University, London, ON Canada; 14 Lawson Health Research Institute, London, ON Canada; 15 Douglas Mental Health University Institute, Department of Psychiatry, McGill University, Montreal, QC Canada; 16 Division of Informatics, Imaging & Data Sciences, University of Manchester, Manchester, M13 PT UK

**Keywords:** Diagnostic markers, Schizophrenia

## Abstract

We investigated the longstanding idea that the onset of psychotic symptoms in schizophrenia arises from an early phase of glutamate neurotoxicity, possibly related to loss of GABA restraint, oxidative stress or inflammation, that cumulatively results in a later phase of synaptic loss in keeping with magnetic resonance spectroscopy (MRS) evidence of reduced glutamate in schizophrenia, especially in older patients. We evaluated this hypothesis in a 3-centre MRS study to determine whether abnormalities in glutamate in dorsal anterior cingulate cortex (dACC) differed between people with minimally treated ‘Recent’ onset schizophrenia and an ‘Established’ group with > 10 years of illness. We tested the hypothesised mechanisms of reduced GABA in either or both dACC and occipital cortex, and depletion of dACC glutathione, a measure of central inflammation. We explored predicted associations between MRS variables, circulating cytokines and clinical symptoms. The Established group showed significantly greater dACC glutamate deficit than the Recent group which was not accounted for by lifetime exposure to antipsychotic drugs or by their greater CRP or IL-6 levels nor was the deficit associated with glutathione depletion. The greater dACC glutamate deficit in established illness is compatible with loss of synapses occurring after onset of symptoms but there was little to suggest underpinning excitotoxicity, inflammation, or oxidative stress. GABA was reduced in patients versus controls across dACC and occipital voxels. Only dACC GABA content correlated significantly with symptoms, lower content with greater positive and negative symptoms across both groups and this is supportive of a pathophysiological role of GABA in psychosis.

## Introduction

The onset of psychosis peaks in early adulthood, and has long been attributed to an exaggeration of neurodevelopmental cortical synaptic pruning, a process which is most active in late adolescence [1–3]. An alternative theory suggests that an initial excess of glutamate release induces psychotic symptoms and leads to neurotoxic damage to synapses which then leads to glutamate deficits and more global functional impairments as the illness progresses [4]. Recent longitudinal imaging studies suggest that loss of medial frontal cortical grey matter may occur within the few weeks preceding of the transition from the prodrome to psychosis [5], which would be consistent with synaptic loss due to excitotoxic damage or excessive pruning in early illness.

Reduced levels of ACC glutamate have been reported in several MRS studies in schizophrenia. A landmark meta-analysis by Marsman et al reported overall increased MRS glutamine in medial frontal cortex (MFC)/anterior cingulate cortex (ACC) but decreased glutamate in studies comparing patients versus controls. However, in a meta-regression they found that both metabolites decreased with age of the cohorts at faster rate than controls [6]. They concluded that the results were consistent with an early excitotoxic process leading to reductions in glutamate. However, they acknowledged other explanations including a possible cumulative effect of exposure to antipsychotic drugs. Subsequent meta-analyses of the expanding MRS literature have generally corroborated MFC glutamate deficits in schizophrenia, but it less clear whether this is age-related [7, 8]. Merritt et al. found age-related declines in MFC glutamate in both patients and controls, but concluded that lower MFC glutamate levels in patients may be better accounted for by antipsychotic exposure than by greater age-related decline [9].

In this three-centre study, we aimed firstly, to clarify the extent to which reduced MFC glutamate in schizophrenia is integral to the illness rather than effects of age by directly comparing patients recruited at contrasting stages of illness. Secondly, we conducted a series of pre-specified secondary and exploratory analyses based on mechanistic hypotheses about the neurobiological origins of such changes in schizophrenia and their clinical consequences.

A key mechanistic aim of the study was to investigate evidence that changes in MRS measures of glutamate might originate from impaired GABA restraint of glutamate neurons and resultant excitotoxic damage as first proposed in the NMDA receptor deficiency hypothesis of Olney and Farber [4]. Direct evidence of GABA deficiency in psychosis exists in reports of reduced levels in postmortem brain and subsequently of impaired synthesis specifically in parvalbumin and somatostatin GABA interneurons that mediate cortical inhibition [10–12]. The discovery that rare autoimmune disorders with antibodies against NMDA receptors can present as schizophrenia was a remarkable corroboration of the NMDA deficiency theory while also reinforcing interest in the neuroinflammatory hypothesis of schizophrenia [13]. Circulating inflammatory cytokine concentrations such as Interleukin-6 (IL-6) and C-reactive protein (CRP) are consistently increased across many cytokine screening studies in schizophrenia [14]. Whether peripheral cytokines penetrate the brain to induce a damaging microglial neuroinflammatory state and grey matter loss [15] or exert direct effects on synaptic development, function and survival is debated [16–18]. We investigated the potential role of inflammation in dACC glutamate abnormalities detected by raised peripheral cytokines and depletion of MRS glutathione, the brain’s principal anti-oxidant, depleted by reactive oxygen species generated by inflammation.

In summary, the primary study prediction was that ACC glutamate would show greater deficits in the established group than in the recent onset group consistent with neurotoxic synaptic loss. We derived measures of current (AP-day) and cumulative (AP-life) exposure to antipsychotic drugs from an examination of contemporaneous case notes to control for their possible influence on ACC glutamate content. In a set of 5 secondary pre-specified hypotheses, we predicted GABA deficiency in patients relative to controls, regardless of phase, and for abnormalities of ACC glutathione, as an index of central inflammation, in either or both phases of illness. Within the patient group, we used correlation analysis to a) explore the potential role of Glutamate and GABA in the pathogenesis of symptoms, and b) to detect the possible associations between demographics, drug exposures and inflammatory markers with the MRS variables. Follow-up mediation analysis determined the degree to which apparent associations could account for group differences in the primary outcome, ACC glutamate.

## Methods

### Ethical statement

The study received ethical approval from the National Research Ethics Service Committee Northwest – Lancaster UK, reference 14/NW/0298 on 18/06/2014. Written informed consent was obtained on the approved form and retained on the site file. All methods and procedures conformed with the relevant guidelines and regulations.

### Participants

Participants with schizophrenia under the care of local psychiatric services were recruited by clinical research fellows who prepared a case-summary for a consensus diagnosis process between 3 clinicians [19]. All patient participants currently met DSM IV criteria for schizophrenia, schizoaffective disorder, or schizophreniform disorder. Two samples of patients (aged 18–55 years and fluent in English) were recruited at each site, from populations representing two phases of illness: a minimally treated group (less than 12 weeks) with recent onset of illness of less than 5 years (Recent group) and a group with established illness of at least 10 years duration (Established group). We confirmed that all participants in the Recent group met criteria for schizophrenia 12 months after MRS data collection. Two groups of healthy Control participants were recruited locally by public advertisement and selected to match the patient groups (site-wise) for age, sex, and parental occupation using the National Statistics Socio-economic classification (NS-SEC), 5-class self-coded method [20]. The intended final sample size was 60 patients (20 per site) for each Phase Group, and 30 healthy controls (10 per site) age matched to each of the patient groups giving a planned total sample of 180. For full inclusion and exclusion criteria see Supplementary Material Section 1.

### Clinical variables

We recorded years of education and body-mass index (BMI) for all participants. Additional measures for patients included duration of illness; current antipsychotic defined daily dose (AP-day); and a 11- point rating of lifetime antipsychotic exposure measure (AP-life) reflecting both dose and duration of medication (obtained from contemporaneous patient records that included medication name frequency dose route and start and end dates) with fixed criteria for each level (Supplementary Material Section 2). Symptom measures included Positive and Negative symptom scores assessed using PANSS [21]. Cognitive function in all participants was assessed using a short-form of the Wechsler Adult Intelligence Scale, 3^rd^ Ed [22]. WAIS-III [23] validated for schizophrenia [24], and consisting of four subtests: Digit-symbol coding; Information; Block Design; and Arithmetic [24]. Subtest scores were combined to give a Full Scale IQ Standard Score (FSIQ). We used the Wechsler Test of Adult Reading (WTAR) as a proxy for pre-morbid IQ [25]. Psychosocial functioning was assessed using the Personal and Social Performance Scale (PSP) [26].

### MR Spectroscopy

Metabolites of interest were glutamate, glutamine, glutathione, and GABA, and were measured in two voxels, one placed bilaterally in the dorsal anterior cingulate cortex (dACC, 35x40x20 mm^3^) and one in occipital cortex (OC, 30x30x30 mm^3^at 3 T, 28 × 28 × 28 mm^3^at 7 T). 3 T scanners were used at Cardiff and Manchester and a 7 T scanner at Nottingham. Short-echo spectra (PRESS TE/TR = 35/2000ms at 3 T, STEAM TE/TM/TR = 17/17/2000 ms at 7 T) were acquired at all sites. At 7 T the STEAM spectra were used to measure all 4 metabolites, while at 3 T MEGA-PRESS was used to measure GABA (TE/TR = 68/2000 ms) and glutathione (TE/TR = 130/2000). The edited GABA signal from MEGA-PRESS is contaminated by co-edited macromolecules [27] and is often referred to as GABA + , though we use GABA to describe the signal. Unsuppressed water acquisitions were acquired from each voxel to provide a reference for absolute quantification and corrected for voxel CSF, which was greater in established patients but unaffected by diagnosis (Supplementary Figure 3.1 and Table 3.1). For full MRS details for each site see Supplementary MRS Methods. MRS measures were referenced to water, consistent with best practice as described in the recent consensus articles on acquisition and processing of human brain 1H MRS data [28, 29]. We removed extreme values lying more than three times the IQR beyond the boundaries of the interquartile range, for that site, and site-normalised all values by subtracting the site median from each value and dividing by the median absolute deviation (MAD). (Supplementary Figure 4.1). Descriptive statistics for sample sizes, raw MRS and cytokine values by site and group are reported in Supplementary Figure 5.1-5.3.

### Cytokines

Venous blood samples taken on the day of imaging were collected and centrifuged within one hour at 1300–2000g for 10 min, and plasma stored at −70 °C degrees at each site prior to shipping in batches for assay. Cytokines were measured using Meso Scale Discovery (MSD) V-plex immunoassays (human) kits (MSD, Rockville, USA) [30, 31]. Any cytokine value below the standard Minimum Detectable Value (MDV) was replaced with the MDV. We log_10_-transformed the positively skewed assayed values, then site-normalised them (as for the MRS values) to control for small but systematic site differences.

### Statistical methods

We used ‘robust’ methods for all descriptive and inferential statistics, implemented in R version 4.1.2 [32] using functions from the WRS2 package (Supplementary Material Section 6) [33]. Robust methods are a family of methods that are robust to deviations from the distributional assumptions of parametric methods (e.g. long-tailed distributions), and to excess leverage from outlying values. For mediation models we used the lavaan structural equation modelling (SEM) package, version 0.68 [34], with the MLR estimator (Maximum Likelihood estimation with Robust standard errors) and removed outlying values prior to analysis. Variables with skewed distributions were transformed to symmetrical distributions (log_10_ for BMI and cytokine values; square root for AP-day).

### Statistical Design, hypotheses and predictions

The study was designed to have 80% power at an alpha of 0.05 to find differences in dACC glutamate abnormality between phases (Phase x Diagnosis interaction). We selected dACC glutamate as the primary measure being more accurately determined than glutamine at 3 T. The expected effect size was based on Marsman et al.’s meta-analysis showing increasing deficits in dACC glutamate with mean sample age with a slope of −0.04 ± 0.03 per year of age [6]. This indicates a medium effect size (Cohen’s d = 0.6) for comparisons between groups differing by 15 years. As the primary analysis, uncorrected *p* values are presented for this test. We also predicted that low GABA in patients would be evident regardless of phase of illness and in either or both dACC and OC voxels, and therefore tested for main effects of Diagnosis in dACC, OC, and the voxel mean. For dACC glutathione, we predicted depletion in early or both phases, tested respectively by Diagnosis x Phase interaction and a main effect of Diagnosis. The analysis plan is summarised in Supplementary Material Section 7. We applied an FDR adjusted alpha correction for these five additional tests, and report both uncorrected *p* values and FDR corrected *q* values (Supplementary Tables 7.1–7.3).

In our funded proposal we made the following set of additional mechanistic correlational predictions:

- Reduced GABA content will be associated with increased dACC glutamate or glutamine or both in Recent onset cases.
- Positive symptoms will be associated with greater dACC glutamate and reduced GABA in Recent onset patients whereas negative symptoms will be associated with low dACC glutamate in established patients.
- In early schizophrenia, depleted dACC glutathione in Recent onset patients will be associated. with increased dACC glutamate or glutamine and increased circulating CRP and IL-6 concentrations.

For these predictions, as well as for all other exploratory analyses, we present uncorrected *p* values as measures of model fit only, taking < 0.05 merely as ‘evidence in support of the hypothesis’ [35, 36].

## Results

### Participant characteristics

There were 62 (15 female) patients and 35 (9 female) controls in the Recent group and 76 (23 female) patients and 39 (14 female) controls in the Established group, slightly exceeding the target sample sizes (Supplementary Table 5.1). Summary statistics and group comparisons for demographic, cognitive, and clinical variables are shown in Table 1. Details on missing or unusable data and are given in Supplementary Tables 5.2 and 5.3. Participants prescribed valproate or phenytoin were excluded. Participants refrained from use of zopiclone (n = 7) and ‘as required’ diazepam< 5 mg/day (n = 4) for 48 h before MRS scanning. Minimal recreational cannabis use was permitted in 8 participants who used more than once a month but abstained for at least 48 h before scanning.

**Table 1:** Demographic, cognitive and clinical variables by group.

	Recent				Established				Patients	
	Controls	Patients	Difference^a^		Controls	Patients	Difference^a^		Difference^b^	
**Demographic**	Mean (SD)		Tr-Diff.	P	Mean (SD)		Tr-Diff.	P	Tr-Diff.	P
Age	24.4 (4.4)	24 (5.1)	−0.60	0.521	42.3 (7.8)	42.4 (7.5)	0.28	0.883	19.6	N/A
Parental class (NS-SEC)	2.7 (1.7)	2.9 (1.6)	N/A	0.635	2.9 (1.6)	2.8 (1.8)	N/A	0.721	N/A	0.750
Years of education	16.5 (2.6)	14.7 (2.3)	−2.16	**0.001**	17 (3.6)	14.5 (3.1)	−2.81	**0.001**	−0.5	0.377
BMI	24.1 (3.6)	25 (4.4)	0.01	0.988	25.8 (4)	29.8 (5.6)	4.40	**0.000**	5.4	**0.001**
Current IQ (FSIQ)	108.9 (22.5)	94.3 (17)	−18.75	**0.000**	105.2 (16.2)	89.9 (14.2)	−15.17	**0.000**	−3.5	0.293
Pre-morbid IQ (WTAR)	106.8 (17.5)	100.3 (18.6)	−8.43	**0.025**	108.4 (9.7)	100 (17.1)	−7.13	**0.017**	−0.1	0.982
FSIQ-WTAR	−2.1 (14.9)	5.9 (15.3)	−8.83	**0.002**	3.1 (14.2)	10.1 (14.8)	−7.88	**0.011**	−5.2	0.054
**Clinical**										
Antipsychotic score										
AP-day (0–4)		0.7 (0.7)				1.3 (0.8)			0.7	**0.000**
AP-life (0–10)		1.7 (0.8)				8 (1.3)			6.2	**0.000**
PANSS Negative		14.5 (6.0)				13.4 (5.3)				0.495^c^
PANSS Positive		12.5 (5.2)				13.2 (6.1)				0.286^c^
PSP		63.5 (14.2)				56.4 (13.5)			−8.1	**﻿0.011**

Tr-Diff is difference between 20% trimmed means. P values are for Yuen’s robust t-test for differences between trimmed means. NS-SEC p values for the linear-by-linear association χ^2^ test). P values <0.05 are shown in bold. WTAR and FSIQ values are Standard Scores (pop. Mean = 100, SD = 15)

*PSP* Personal and Social Performance

^a^Patient-Control difference

^b^﻿Recent -Established difference

^c^p value computed using log_10_-transformed scores

Mean scores on all cognitive measures were lower in the patient groups than in their age-matched control groups and they had fewer years of education. Decline from premorbid IQ (FSIQ-WTAR difference) was greater for both patient groups than their controls. The Recent and Established patient groups did not differ appreciably on years of education nor cognitive test scores, but the Established group had a lower mean PSP score. Decline from premorbid IQ (FSIQ-WTAR) was slightly greater in the Established Group. Patient groups did not differ appreciably in severity of Positive and Negative symptoms. Daily antipsychotic dose and mean cumulative antipsychotic exposure were both greater in the Established group. Mean BMI was also greater in the Established than in the Recent patients, as well as higher than their age-matched control group.

### Glutamate and GABA: effects of Diagnosis and Phase

**Fig. 1:**
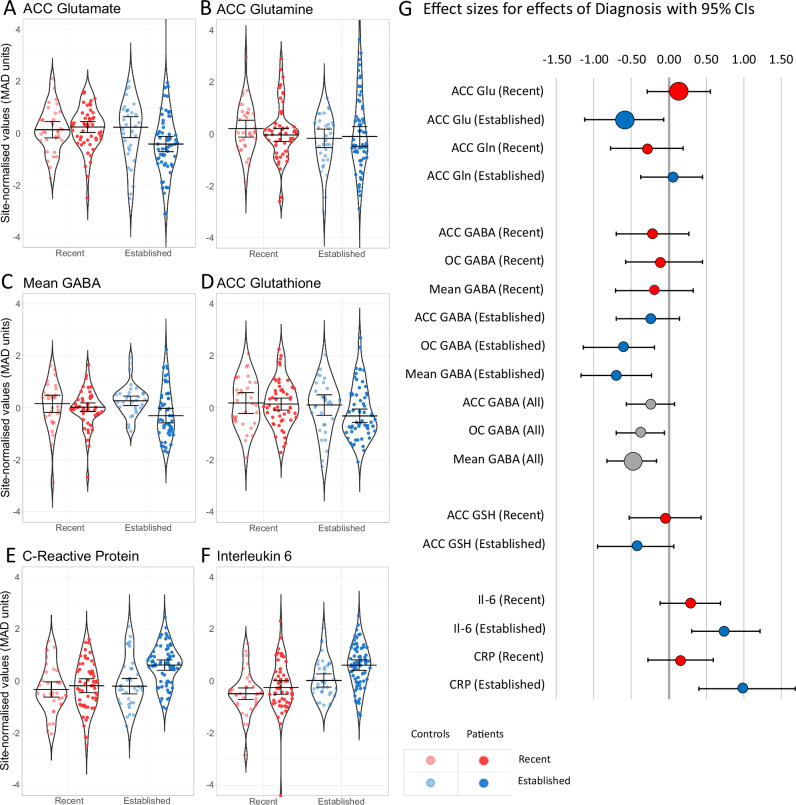
Effects of Diagnosis and Phase on MRS variables and cytokines. Effects of Diagnosis for MRS variables (**A** to **D**) and inflammatory cytokines (**E** and **F**). Panel G shows robust effect sizes (AKP, a robust version of Cohen’s d) for effects of diagnosis, with 95% CIs. Supplementary Table 6.2 shows robust effect sizes and CIs for effect of diagnosis on all variables in each group.

Greater reductions in dACC glutamate were found in Established patients than in the Recent group relative to their age-matched controls, as predicted, and evidenced by a statistically significant Diagnosis x Phase interaction, Q = 5.74, N = 183, *p* = 0.022 (Fig. 1A). There were no significant interactions with Site. Follow-up tests found no evidence of elevated glutamate concentration in the Recent onset group (ES = 0.13 (95% CI: −0.32, 0.59), *p* = 0.574 but there was a large effect size for the reduction in dACC glutamate in the Established group relative to controls, ES = −0.59 (95% CI: −1.14, 0.09), *p* = 0.012. An exploratory analysis of dACC glutamine showed no evidence of a Diagnosis x Phase interaction (Fig. 1B), *q* = 0.60, N = 180, *p* = 0.440.

In keeping with a generalised GABA deficit in psychosis, average GABA concentrations (dACC & OC) were lower in patients (Fig. 1C), Q = 8.08, N = 155, *q* = 0.036, *p* = 0.006), with a Medium ES = −0.48 (95% CI: −0.83, −0.16) for the effect of Diagnosis (Fig. 1G). There was no evidence for interactions between Diagnosis and either Phase (*p* = 0.102), or Voxel (*p *= 0.999, as tested by a 3-way ANOVA with dACC-OC difference as the dependent variable). There were no significant interactions with Site.

### Glutathione and Cytokines

dACC glutathione was not depleted relative to controls overall (*q* = 0.296, *p* = 0.197), nor was there any Phase x Diagnosis interaction (*q* = 0.350, *p* = 0.175) or any interactions with Site. Exploratory ANOVAs for CRP and IL-6 concentrations (Fig. 1E and F) showed a similar pattern of greater elevation relative to controls in Established than in Recent onset groups. There was evidence for a Diagnosis x Phase interaction for CRP (*p* = 0.047) but not for IL-6 (*p* = 0.159), indicating higher mean CRP, relative to controls, in the Established than in the Recent patient group. For both cytokines there was evidence of a main effect of diagnosis (CRP *p* = 0.004; IL-6 *p *= 0.003), indicating higher values in patients than controls across both groups.

### Correlation analysis of mechanistic markers and confounders

**Fig. 2:**
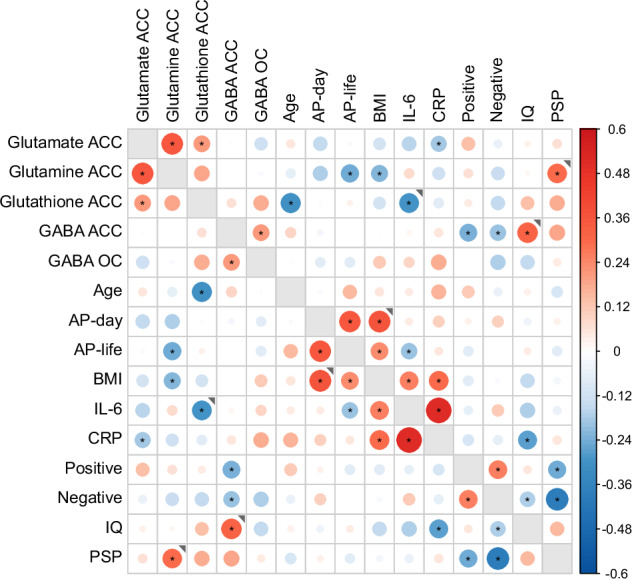
Correlation matrix for MRS variables, potential mediators, and clinical correlates. Correlation matrix relating 4 MRS dependent variables (top left) for all patients (n = >100) to potential mediators and to clinical correlates. Size and colour intensity indicate magnitude of correlation, red for positive and blue for negative correlations scaled from −0.6 to + 0.6 using group mean centred data. Asterisks indicates all *p* values <0.05. Correlations are across both groups except where the grey marker ◥ indicates that the correlations differed between groups at p < 0.01 and was *p* < 0.05 only in the Recent onset group. There were no instances where the correlation was greater and* p* < 0.05 only in the Established group. Where groups correlations differed but both at *p* < 0.05 in the same direction, the overall average is shown.

To evaluate the pre-specified mechanistic correlational predictions, robust bivariate correlations were computed between the variables of interest together with potential influencing variables, both within patient groups, and, using group mean-centred data, across both patient groups. Correlation coefficients are illustrated in Fig. 2 and shown numerically in Supplementary Table 8.1 and as scatterplots is Supplementary Figure 8.1.

We examined the three sets of predictions specified above. a) There was no evidence of an inverse relationship in the Recent group between dACC GABA and glutamate or glutamine content. b) In terms of symptoms, dACC glutamate did not correlate with positive symptoms in the Recent group or with negative symptoms in the Established group. However, low dACC GABA was associated with positive symptoms across both groups (*r* = −0.24, *p* = 0.014) as predicted for the Recent group but not specified for the Established group. Low dACC GABA also showed evidence of association with negative symptoms across both groups (*r* = −0.20, *p* = 0.040) which was not predicted. c) In terms of early inflammatory mechanisms in the Recent group, there was a negative correlation between dACC glutathione and IL-6 (*r* = −0.30,* p* = 0.037) concentrations, but not CRP. dACC glutathione and both dACC glutamate and glutamine showed positive correlation rather than negative.

The correlation matrix overall indicated a number of patterns of association between variables that might either confound, or account for, the greater dACC glutamate deficit in the Established group. dACC glutamate, glutamine and glutathione content were all positively intercorrelated (Fig. 2 top left), while CRP and IL-6 levels tended to be inversely correlated with dACC glutamate across groups. Neither age nor duration of illness correlated with dACC glutamate across groups (r = ≤ 0.10; Supplementary Table 8.1). There was no indication that either AP-day or AP-life were associated with lower dACC glutamate. AP-life showed its expected association with higher BMI, which in turn showed its well-known association with increased cytokines (CRP and IL-6). Nevertheless, AP-life showed an association with lower IL-6 concentrations (*r* = −0.20, *p* = .023), suggesting a possible anti-inflammatory effect that might mask illness-related increases in IL-6 that could mediate the changes in dACC glutamate. Similarly, evidence indicated that AP-life was associated with decreased dACC glutamine, despite no evidence of lower dACC glutamine in the Established group.

### Mediation analysis of the dACC glutamate deficit in Established patients

The correlation analyses (Fig. 2 above) identified several possible influences that might partially or wholly account for the greater dACC glutamate deficit in Established patients. These included effects related to AP medication exposure, but also potential mediation by co-occurring phase differences in circulating inflammatory cytokines (CRP and IL-6). We evaluated these possibilities in a sequence of mediation models in which we expressed the values of MRS and inflammatory variables as deviations from their age-matched control means (adjusted variables denoted by Δ).

**Fig. 3:**
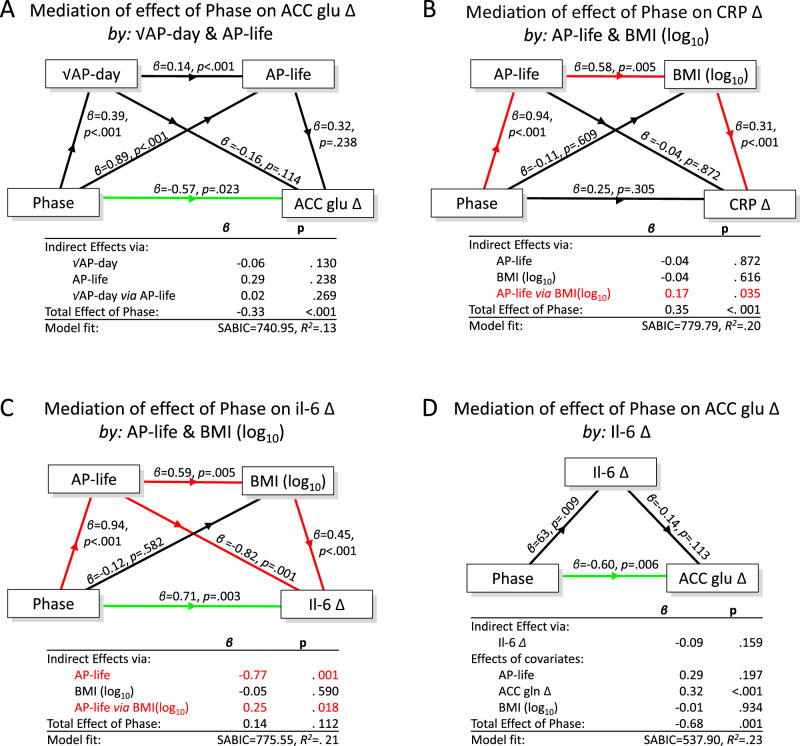
Mediation analysis of phase-dependent loss of dACC glutamate. Models A to C: medication-related mediators of Phase effects on dACC glutamate, CRP and IL-6. Model C revealed excess IL-6 in the Established group (*p* = .003 for the Direct effect of Phase) after accounting for medication-related effects. Model D: Phase-related increases in IL-6 did not mediate the phase-related reductions in dACC glutamateΔ (*p* = 0.159 for the Indirect Effect). Coefficients are standardised *β* values. Total Effect = sum of direct and indirect effect of Phase. Indirect pathways are shown in red if *p* < 0.05. Direct non-mediated effect of Phase shown by horizontal connector, green if *p* < 0.05. Note that in Model D, the Total Effect excludes indirect effects mediated by the covariates.

There was no evidence that the negative Phase effect on glutamateΔ (deficit greater in the Established group) was mediated by daily and/or lifetime antipsychotic dose (Fig. 3A); none of the indirect effect pathways reached *p* < 0.1 and the direct (unmediated) effect of Phase on dACC glutamateΔ remained evident (green line, Fig. 3A), *β *= −0.57, *p* < 0.023. However, as the correlation analyses indicated a negative correlation between AP-life and dACC glutamine, we also investigated mediation via the effects of AP-life on dACC glutamineΔ (Supplementary Figure 9.1panel B). We found weak evidence (*p* < 0.1) for partial mediation of the negative Phase effect on dACC glutamateΔ by AP-life via a negative effect on dACC glutamineΔ (*β *= −0.18, *p* = 0.066). However, this effect was offset by an indirect positive effect of Phase on dACC glutamineΔ itself (*β* = 0.19, *p* = 0.059), and accounting for these indirect effects strengthened the evidence for the direct effect of Phase on dACC glutamateΔ (*β* = −0.66, *p* = 0.002)

The total Phase effect on CRPΔ (greater in the Established group; *p* < 0.001) was completely mediated by the indirect pathway from Phase via AP-life and BMI (Fig. 3B and Supplementary Figure 9.1 panel C) leaving no direct i.e. unmediated effect (p > 0.1). However, for IL-6Δ, while AP-life acted via BMI to increase IL-6Δ, this was offset by the negative effect of AP-life on IL-6Δ itself, as noted in the correlation analysis, which was independent of BMI (Fig. 3C red diagonal). Accounting for these opposed indirect effects of AP-life revealed evidence for an underlying direct effect of Phase on IL-6Δ (*β* = 0.71, *p* = 0.003), namely a residual excess of IL-6Δ in the Established group over levels in the Recent onset group, possibly indicative of a Phase-dependent effects of illness.

We investigated whether the revealed effect of Phase on IL-6Δ levels might mediate the dACC glutamateΔ Phase-effect. The Phase effect on IL6 was isolated (Fig. 3D, *β* = 0.47, *p* = 0.054) by covarying the opposing indirect effects of AP-life and BMI shown in Fig. 3C. By including glutamineΔ in the covariates, we controlled for its previously mentioned indirect influences on the effect of Phase on glutamateΔ. This model provided no evidence to support an indirect (mediating) effect of excess IL-6Δ on Phase-associated dACC glutamateΔ (Fig. 3D, *β* = −0.10, *p* = 0.11) and the direct effect of Phase remained evident (β = −0.61, *p* = 0.01). See Supplementary Section 9 for a full account of the mediation model series).

## Discussion

This study aimed to investigate whether patients with established schizophrenia would show greater dACC glutamate deficits than recent onset patients, controlling for potential effects of antipsychotic medication exposure, and to elucidate their mechanistic origins and their role in clinical pathogenesis. The hypothetical framework was that excessive glutamate neurotransmission in early psychosis underpins the emergence of positive symptoms and results in loss of presynaptic glutamate terminals and the development of persistent negative symptoms in established illness. We confirmed with well-matched samples recruited explicitly from patients with early and established illness, that the Established group had greater dACC glutamate deficits but found no evidence of excess glutamate or glutamine in the Recent group. There was a generalised decrease of GABA content across dACC and OC. We found no evidence of dACC glutathione abnormalities in patients. In inverse contrast to dACC glutamate, circulating cytokine concentrations of CRP and IL-6 were greater (significantly for CRP) in established illness than controls raising the possibility of a causal inflammatory process.

In the Recent onset patients, there was no increase in glutamate or glutamine in dACC and neither correlated with positive symptoms as we had predicted. Our finding is in keeping with the most recent meta-analysis that found no change in MFC glutamate in 12 studies in drug naive patients [7]. Furthermore, longitudinal studies of anti-psychotic treatment effects on ACC glutamate mostly report that baseline differences from controls [37, 38] or within the patient group, do not change significantly over a minimum of 6 weeks treatment to 6 months or more [39, 40]. It seems unlikely that minimal drug treatment obscured group differences in dACC glutamate in the Recent onset group.

The lack of MRS dACC changes in the Recent onset group in this or other studies does not amount to a decisive refutation of the glutamate neurotoxicity hypothesis because the relationship of static MRS glutamate to synaptic status is too uncertain and the time of onset of the putative glutamate excess relative to psychosis onset is unknown. Nevertheless, it can be noted that was little evidence of ongoing oxidative stress in Recent onset patients; there was no depletion of glutathione nor were levels of circulating cytokines markedly increased as might have been expected had there been inflammatory neurotoxicity undetected by MRS dACC glutamate or glutamine. However, IL-6 was elevated relative to controls across groups and dACC glutathione was negatively correlated with IL-6 (Fig. 2) in the Recent group, a possible hint of early inflammatory central oxidative stress. As previously reported in the Manchester subset, there was no evidence of central microglial inflammation in Recent onset patients as dACC TSPO radioligand binding was not increased in Recent or Established patients [41].

The greater dACC glutamate deficit in the Established group, although in keeping with the Marsman meta-analysis [6], is at odds with the larger mega-regression by Merritt et al. [9], which found no evidence that age-related decline in MFC glutamate was greater in patients than controls. While the meta-analytic literature generally confirms MFC glutamate deficits in patients with schizophrenia [6–9], it remains inconclusive regarding its time course. As effects of illness progression may be substantially more non-linear than effects of age, our explicit comparison between patients at contrasting phases of illness, may have been better placed to find such effects.

In their mega-regression, Merritt et al. [9] found that in a model with 276 patients, antipsychotic dose was negatively associated with MFC glutamate, with age-related decline accounting for additional variance, and possibly accounting for the observed MFC deficits in schizophrenia. However, neither our AP-day nor AP-life drug exposure measures contributed to the phase-related reduction in dACC glutamate in our mediation analysis (Fig. 3A and Supplementary Figure 9.1 panel B). The validity of the AP-life measure is supported by its association (Fig. 2), replicated across both groups, with BMI and with lower IL-6 levels consistent with metanalytic evidence [42, 43]. Therefore, the contrasting association of AP-life with dACC glutamine and not with the other MRS measures (all r = <0.10) could warrant attention to a drug action on glutamate-glutamine cycling through astrocytes influencing glutamate neurotransmission in a way not visible to static MRS. While it seems that drug exposure does not account for the greater deficit of ACC glutamate in the Established group, an influence of antipsychotic drug exposure on glutamate neurotransmission via glutamine cannot be ruled out.

Reduced dACC glutamate in the Established but not the Recent group could point to a range of potentially abnormal process in schizophrenia such as altered glutamate synthesis, turnover, reuptake or astroglial function other than the proposed accumulation of damage to glutamate synapses in established schizophrenia [4, 6, 44]. However, recent PET studies reported that the presynaptic marker SV2A was reduced in patients with established illness but barely in first episode patients [45, 46]. Critically, SV2A correlated with MRS dACC glutamate content (r = 0.55) in healthy controls and in patients in keeping with the predominance of glutamate synapses in cortex and suggesting that MRS glutamate is partly reflective of the presynaptic glutamate pool. Whether the loss involves excitoxicity remains open in the absence of increased glutamate or glutamine in the Recent group. Furthermore, the contribution to pathogenesis is unclear since reduced glutamate did not show the predicted correlation with negative symptoms (table 2) nor was this observed in a metanalysis of 15 studies with age-related decrease in MRS creatine-scaled glutamate [9]. Indeed, the meta-analysis found that against the background of reduced levels, glutamate reliably but weakly correlated with positive symptoms which we did not observe in our sample (Fig. 2).

We evaluated the possibility that previous or current inflammation contributed to the reduction in dACC glutamate in Established cases. Circulating CRP and IL-6 concentrations were increased in the Established group and were weakly predictive of reduced dACC glutamate (Fig. 2). However, mediation analysis indicated that whereas the late phase increases in CRP were accounted for by associated drug treatment and BMI differences (Fig. 3B), the effect of Phase on IL-6 was enhanced after adjustment for treatment confounds (Fig. 3C). Nevertheless, there was no evidence that IL-6 increases directly mediated the late phase decrease in dACC glutamate (Fig. 3D).

There is consistent evidence for impaired GABA synthesis in cortical interneurons in samples of post-mortem brain in schizophrenia, but it is less clear whether GABA neurones are lost [11]. The interpretation of in-vivo MRS GABA studies is complicated by variable definitions of ROIs and by clinical and treatment status. In 17 MRS studies with ROIs ranging from subgenual, pregenual, anterior, and dorsal cingulate, found no overall decrease in GABA [47]. More recently, Simmonite et al reported an overall decrease in GABA in 10 studies in ROIs corresponding to our dACC ROI [48]. The authors suggest GABA deficits may be modulated by illness stage (greater in acute) or ameliorated by prolonged medication. However, the acute-chronic difference was small and not statistically significant. We found no evidence for an effect of phase or of medication exposure on GABA deficits in patients. Iwata et al. [49] found 3 studies in OC all 3 reporting reductions in GABA in keeping with our study [49]. Our MRS findings are strongly corroborative of a report of lower GABA concentrations measured directly in lumbar cerebro-spinal fluid in 40 prospectively recruited patients with FEP and 21 controls [50]. Furthermore, reduced CSF GABA correlated with PANSS positive scores and functional outcomes as in our study in which positive and negative symptoms correlated with low GABA across Recent and Established groups. The findings suggest that low GABA may have a key role in the pathogenesis of symptoms in schizophrenia but not apparently acting via glutamate disinhibition.

We acknowledge our results and inferences apply to dACC and OC and may differ in the many other brain regions examined in numerous individual studies. For example, Nakahara et al [8] meta-analysed 134 studies in 14 brain regions with 6 different configurations of patient group including drug-free. While some themes emerge such as the overall reductions in glutamate and GABA in some comparisons in medial frontal cingulate regions there is significant heterogeneity in other comparisons. In view of the limitations of static MRS measures, further understanding of synaptic pathology in schizophrenia using MRS will depend on the development of high resolution and dynamic paradigms such as drug or cognitively-evoked MRS responses [51] and dynamic glutamine-glutamate turnover using 13C-MRS. With new MRS methods in conjunction with MRI and MEG measures of connectivity, computational models of synaptic dysfunction in schizophrenia should become possible [52].

## Limitations

This is a cross-sectional study and therefore we cannot conclude that differences between the Recent onset and Established cases arise from longitudinal change within cases. It is possible that there was an accumulation of cases with more persistent or severe illness, associated deficits in MRS metabolites and higher cytokine levels in the Established group. Although the overall clinical sample is large, the Recent and Established groups were designed to have half the number of controls (n = 30) as patients (n = 60) to increase the power of within-patient analyses but at the cost of power for control- patient comparisons. The multi-site design and will have introduced noise owing to differing clinical and healthy populations sampled and different MR field strengths compounded by the lack of calibrating phantoms or travelling heads. Furthermore, although MRS procedures and analyses were co-ordinated, they were not completely harmonised in order to take advantage of local expertise and manpower, as detailed in Supplementary MRS Methods. This may have contributed to site effects in metabolite quantification, not completely controlled for by statistical procedures explained in Supplementary Section 4. In common with most studies, we used GABA MEGAPRESS without macromolecular suppression to avoid inconsistencies arising from the uncertain effects of small field drifts on MM suppression besides the lack of definitive evidence that MM suppression improves GABA detection or change. MRS measures of glutamate and GABA content offer uncertain and limited information about the state of glutamate and GABA neurotransmission. Static MRS measures are the highly uncertain sum of signals from various intracellular pools of glutamate/GABA not all of which are visible to MRS [53]. Synaptic and extrasynaptic pools are thought respectively to be too small or dilute to contribute signal [54]. Interpretation of group differences requires correlated measures of origins and functional consequences.

## Conclusions

In investigating the neurotoxic synaptic loss account of schizophrenia pathogenesis, we confirmed the prediction that MRS glutamate deficits in anterior cingulate cortex are greater in established than in recent-onset schizophrenia. The deficit appears to be the result of a process which is not apparent in early-stage illness, and did not appear to be mediated either by current or cumulative antipsychotic exposure. There was no evidence that dACC glutamate deficits were mediated by the greater IL-6 or CRP levels in the Established group nor by oxidative stress. Overall, there was little to suggest underpinning neurotoxicity, inflammation, or oxidative. There were no associations between dACC glutamate and symptoms to suggest a role in clinical pathogenesis. GABA content was reduced compared to controls across anterior cingulate and occipital cortex and across the full sample of patients with recent or established schizophrenia. Lower dACC GABA content was associated with greater positive and negative symptom severity, but did not correlate with markers of inflammation, degree of drug treatment or with glutamate. These in-vivo findings strengthen the evidence that reduced GABA levels are involved in the pathogenesis of the symptoms of schizophrenia.

## Supplementary information

Supplementary Material

Supplementary MRS Methods

## Data Availability

The database for this study database will be made available on reasonable request to bill.deakin@manchester.ac.uk.
