# Supplementary material for: Investigating the disturbance in cortical glutamate and GABA function in psychosis and its origins and consequences
Source: Mol Psychiatry. 2025 Nov 19;31(4):1983–91. doi: 10.1038/s41380-025-03337-x (PMC12999488; doi:10.1038/s41380-025-03337-x)
Supplement: Supplementary file 2 — Supplementary MRS Methods [file 41380_2025_3337_MOESM2_ESM.pdf]

Supplementary MRS methods

Acquisition details

Acquisition details for each site are contained in the following Tables, which encompass the information suggested in the consensus article on reporting of MRS studies (1):

- Table S1 Scanners and Coils
- Table S2 Sequence Parameters and Processing Software
- Table S3 Voxel sizes and locations
- Table S4 Acquisition parameters
- Table S5 MEGAPRESS acquisition details
- Table S6 T_1-weighted anatomical image acquisitions

Table S1: Scanners and Coils

Site	Scanner	Field	Receive Coil
Cardiff	GE HDx	3.0T	8 channel head coil
Manchester	Philips Achieva	3.0T	32 channel head coil
Nottingham	Philips Achieva	7.0T	32 channel head coil

Table S2: Sequence Parameters and Processing Software

Site	Sequence	TE/(TM)/TR ms	Aves	Processing
Cardiff / Manchester	PRESS[a]	35/2000	128	jMRUIv6 QUASARY
Cardiff / Manchester*	GABA MEGAPRESS[b]	68/2000	300/296*	GANNET 2.0
Cardiff / Manchester	GSH MEGAPRESS[c]	130/2000	256	jMRUIv6 AMARES
Nottingham	STEAM[d]	17/(17)/2000	288	LCModel

Citations: a(2); b(3, 4);c(5);d(6)

Table S3: Voxel Sizes and Locations

Site	Voxel Location	Voxel Size mm
Cardiff / Manchester / Nottingham	Bilateral ACC	35x40x20
Cardiff / Manchester / Nottingham*	Occipital Cortex	30x30x30 / 28x28x28*

Table S4: Acquisition details

Site	Sequence	BW (Hz)	pts
Cardiff	PRESS	2000	1024
Cardiff	MEGAPRESS	5000	4096
Manchester	PRESS / MEGAPRESS	2000	1024
Nottingham	STEAM	4000	4096

Table S5: MEGAPRESS Acquisition (Cardiff, Manchester)

Metabolite	TE (ms)	Frequency offsets (edit/control)	Edit pulse shape	Length/BW
Glutathione	130	4.56/1.44 ppm	gauss	14ms/106Hz
GABA	68	1.90/7.6 ppm	gauss	14ms/106Hz

Table S6: T_1-weighted anatomical images

	Sequence	TI/TE/TR (ms)	Matrix	Voxel size
Cardiff	FSPGR*	450/88/3.5	256x256x192	1mm isotropic
Manchester	IR-TFE*	1150/3.9/8.4	256x256x90	0.9x0.9x1.9mm
Nottingham	IR-TFE*	987/3.4/7.3	256x256x180	1mm isotropic

*3D Fast Spoiled Gradient-Recalled acquisition; Inversion Recovery prepared Turbo (rapid) Field Echo (MPRAGE (7))

Processing of Spectra

Spectroscopic analysis was divided among the 3 centres in order to make best use of the human resources provided by the funding agency and to take advantage of local expertize, such as Cardiff's experience in measuring GABA at 3T, Manchester's experience in processing short-echo PRESS and glutathione MEGAPRESS at 3T and the expertize in Nottingham in 7T MRS. Three processing packages were used: GANNET (8, 9); jMRUI (10-13); LCModel (14). Thus MEGAPRESS GABA data were quantified in Cardiff using GANNET (15), PRESS TE35 and glutathione MEGAPRESS were quantified in Manchester using jMRUIv6 (QUASARY and AMARES respectively(16)) and in Nottingham spectra were quantified using LCModel (17). Each centre used established protocols as detailed further in the cited articles.

T_1-weighted anatomical images were used for accurate voxel placement and image segmentation (Table S6, Figure S1). Cardiff and Manchester images were segmented in Cardiff using GANNET 2.0, while Nottingham images were segmented in Nottingham using SPM8, in both cases based upon Ashburner's and Friston's method (18).

Water referencing

Non water-suppressed acquisitions were acquired in each centre. Using the tissue water signal as reference, molar metabolite concentrations were calculated after correction for the CSF content of the voxel. In Nottingham a tissue specific correction of the water signal was used following Gasparovic et al (19), whereas in Cardiff and Manchester the 'standard' correction employed by LCModel was used which does not account for differences in grey matter and white matter within the spectroscopic voxel. While this will affect absolute values, as the tissue content was not significantly different across groups or centres, comparisons of concentration remain valid.

Fig. S1 Voxels in ACC and OC overlaid in yellow on anatomical T_1-weighted images. Subject was chosen from Manchester.

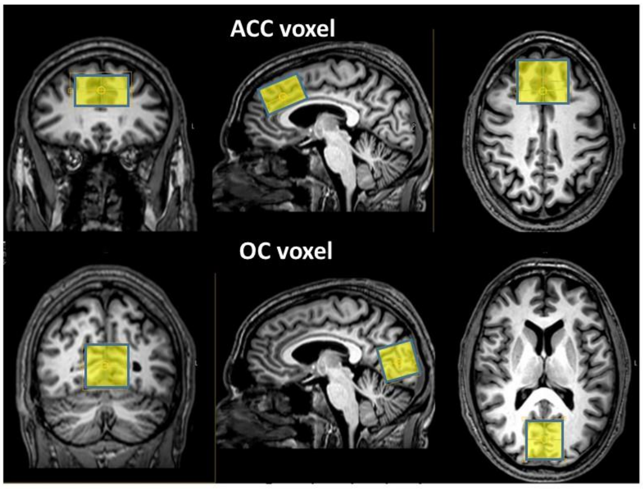

Quality of Spectra

Figs. S2 and S3 show all the short echo spectra that were analyzed from each centre aligned and overlaid on one plot for each voxel location.

Fig S2/S3 Overlay of spectra from ACC and OC. All spectra were loaded into jMRUI v7.0, frequency aligned, residual water removed and intensities normalized by the tissue water signal. No line-broadening was applied for display. Assignments: NAA – N-acetylaspartate, Glut – glutamate; Crn – creatine + phosphocreatine; Cho – choline-containing compounds; m-Ino – myo-inositol

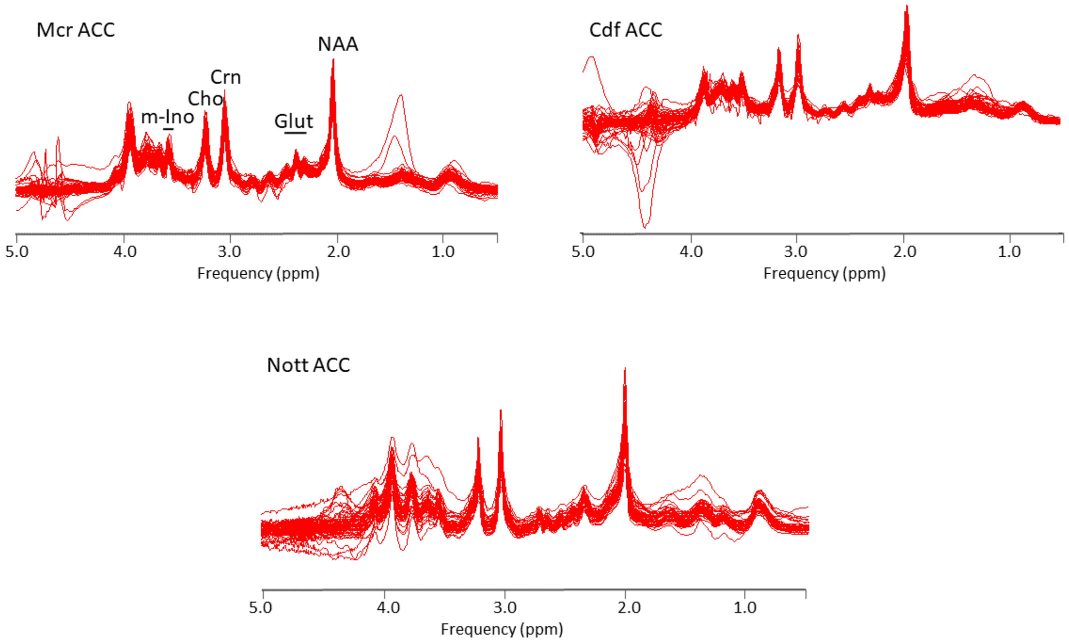

Fig S3 Overlay of short echo spectra from OC. Processed as above.

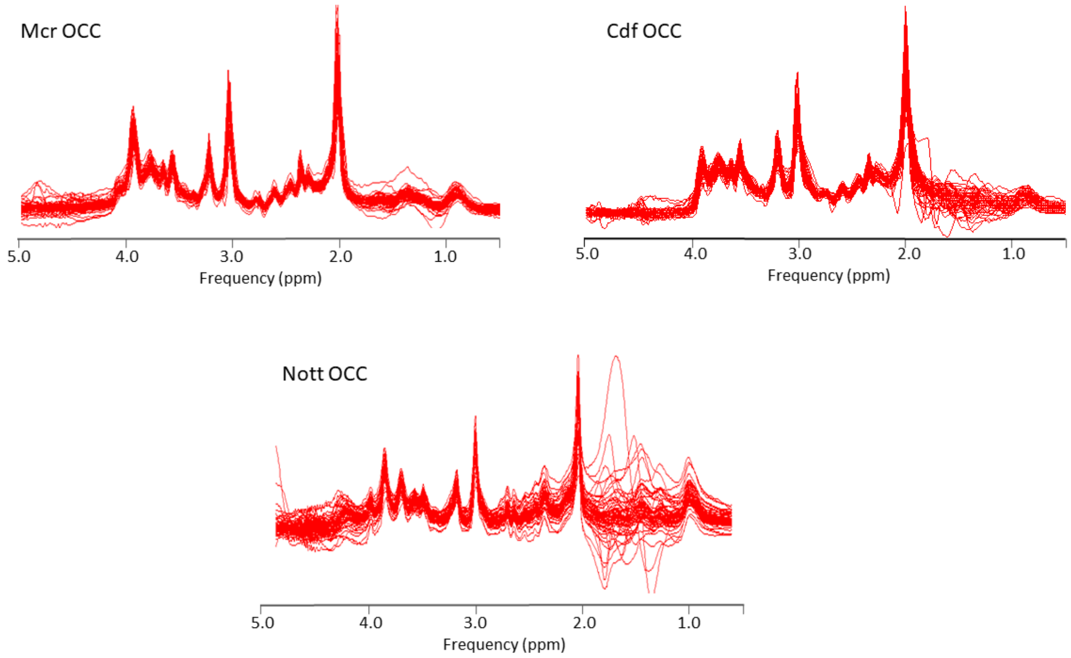

Most spectra from the ACC are free of any significant lipid contamination or obvious artefacts arising from poor water suppression, whereas in the OC the 7T spectra show a number of examples with lipid contamination, though this does not affect the spectra between 2 and 4 ppm. In general the appearance of the spectra is highly consistent with high signal-to-noise ratio and well resolved resonances.

MEGAPRESS spectra from the ACC of all analysed subjects are shown in Fig.S4 for both GABA and glutathione sequences. The relatively long echo times (68ms for GABA, 130ms for glutathione) combined with the low concentration of the metabolites mean the spectra have worse signal-to-noise than the short echo spectra, but in all cases the GABA and glutathione resonances (centred at 3.02 and 2.98 ppm respectively) are quantifiable.

Fig.S4 Overlaid MEGAPRESS spectra from Manchester and Cardiff. Spectra were frequency aligned, line-broadened for display (6Hz) and the intensities normalized to tissue water. Assignments: NAA – N-acetylaspartate; GABA⁺ - edited GABA plus co-edited macromolecules; GSH – edited cysteinyl residue of glutathione; (N) asp – edited aspartyl resonances of NAA, aspartate and N-acetylaspartylglutamate

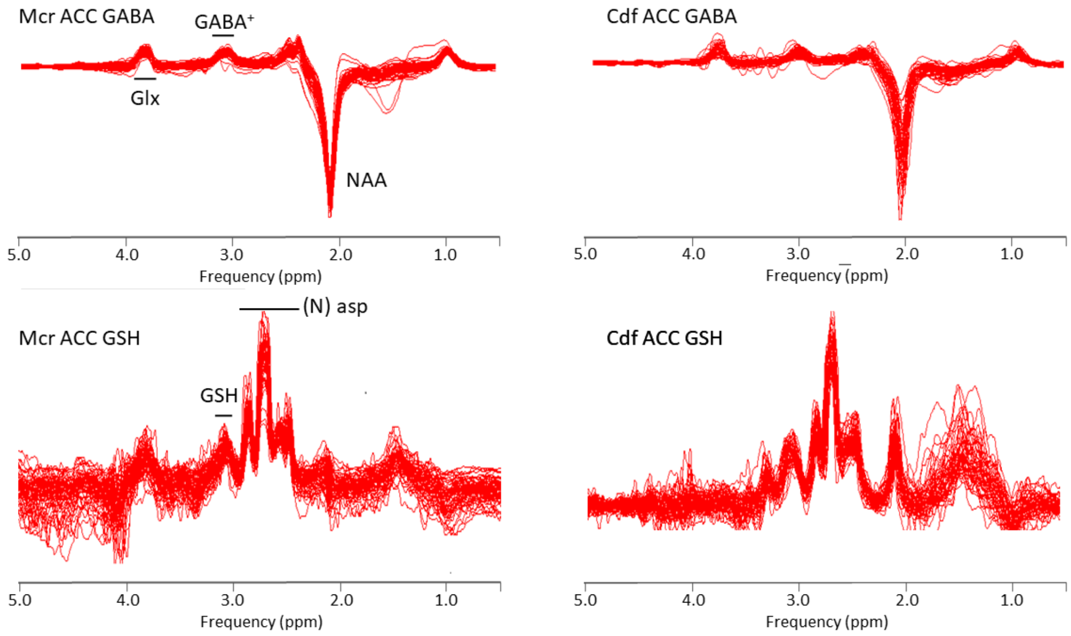

Quantification Results

Examples of the output of the fitting routines for the short echo spectra are shown below for data from Cardiff and Manchester (Fig.S5) and Nottingham (Fig.S6).

Fig. S5 jMRUI fit to Cardiff and Manchester ACC spectra. QUASAR-Y was used to fit the data, with the baseline estimated using the Subtract-QUEST option. The raw data and the estimate are shown in the bottom trace; the estimate with the baseline in the second trace, with individual components and the residual in the upper two traces. A basis set of 6 metabolites and 2 macromolecule components was fitted to the data.

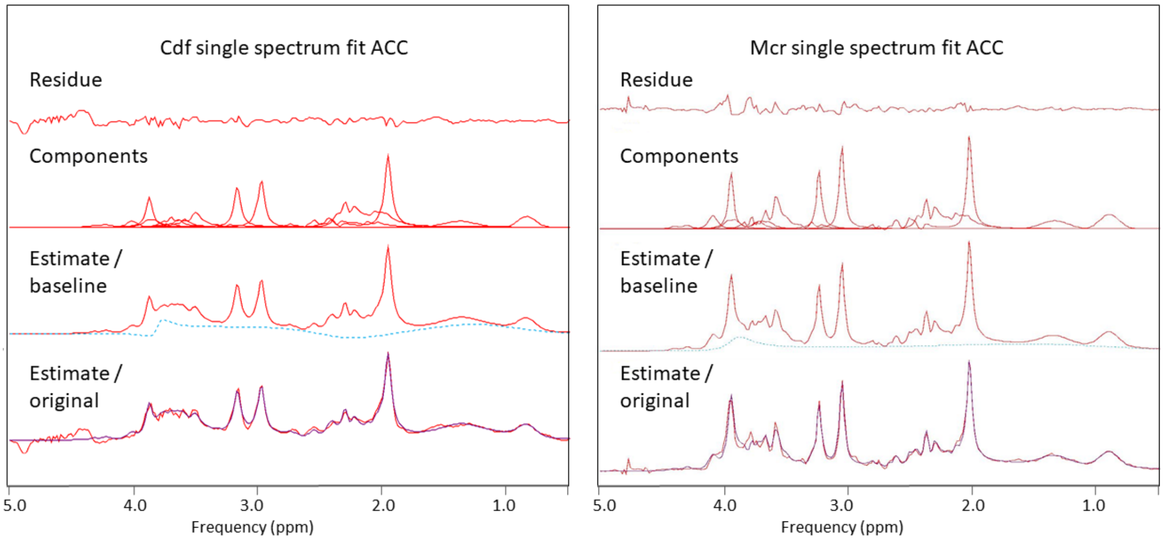

Fig S6 LCModel output for example ACC and OC spectra from Nottingham. Individual fitted components for glutamine, glutamate, GABA and glutathione are shown in the lower traces, together with the residual (bottom) and the original data overlaid on the fit (top). 20 metabolite components plus a macromolecular baseline were fitted to the data.

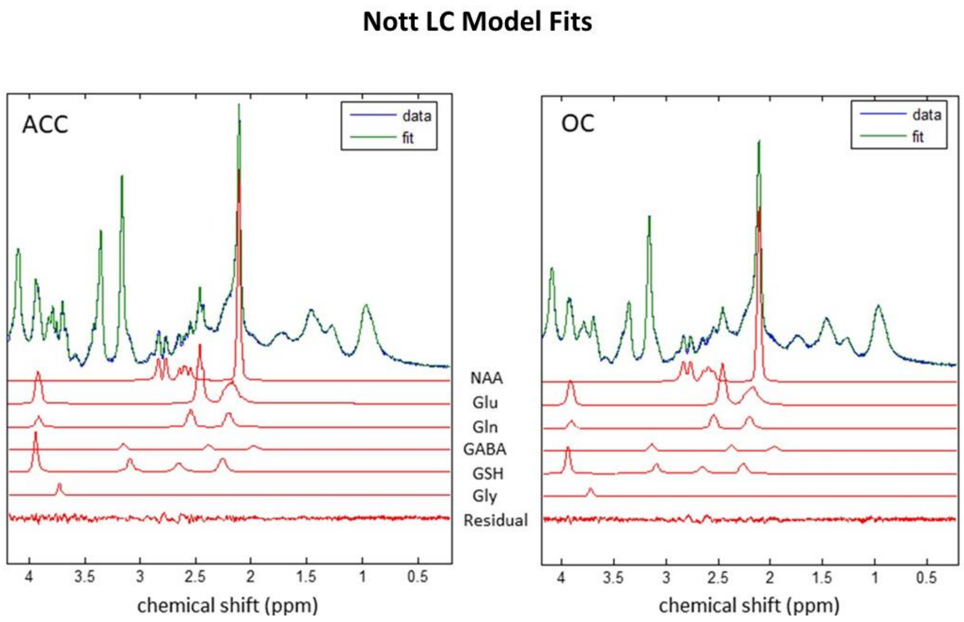

The edited 3T MEGAPRESS spectra were quantified using GANNET in Cardiff for the GABA data and in Manchester using jMRUIv6 for glutathione. Examples of the output of the fitting routines are shown in Figs.S7 (glutathione/AMARES) and S8 (GABA/GANNET).

Fig.S7 AMARES output from glutathione analysis. The glutathione cysteine resonance centred at 2.98 ppm was fitted as a doublet following Kaiser et al. (20), the aspartyl resonances of N-acetyl aspartate were also modelled to improve baseline definition around 3 ppm. The estimate and original data are shown in the bottom trace with the fitted glutathione components and residual above.

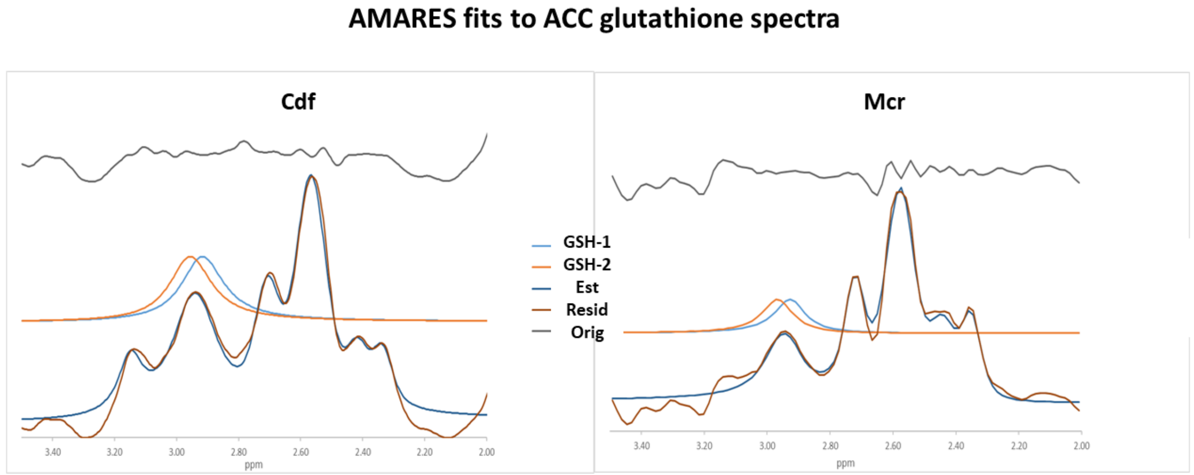

Fig.S8. Examples of spectra fitted by GANNET from Cardiff and Manchester.

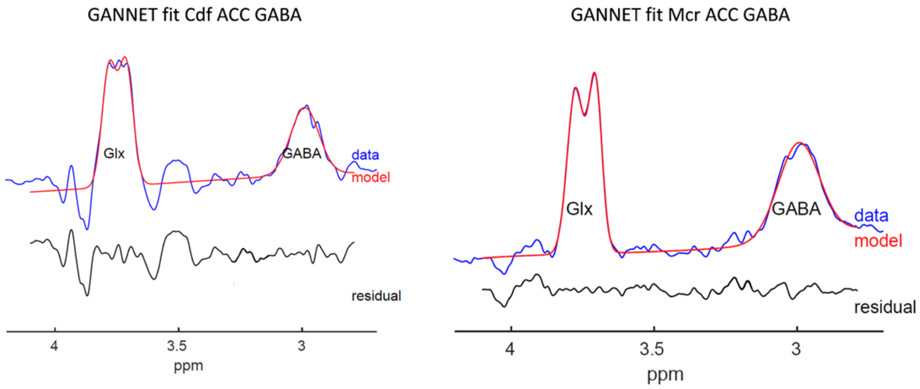

Quality Assurance Metrics

In accordance with recent guidelines on reporting MRS data (1) the following metrics are reported for the short echo spectra in Table S7: water and N-acetylaspartate (NAA) linewidth and %CRLB for NAA and glutamate.

Table S7. Quality Metrics for short echo spectra

Site / brain region	Water LW (Hz)		NAA LW (Hz)		NAA %CRLB		Glut %CRLB	
	ACC	OC	ACC	OC	ACC	OC	ACC	OC
Cardiff	9.3±2.0	9.0±3.2	8.8±2.1	9.2±2.0	0.29±0.14%	0.23±0.05%	0.78±0.47%	0.77±0.39
Manchester	6.8±0.7	6.9±0.5	9.6±1.2	9.7±0.9	0.24±0.07%	0.22±0.001%	0.61±0.18%	0.80±0.30
Nottingham	12.3±1.3	13.6±1.9	15.1±3.4	14.9±2.2	1.06±0.42%	1.94±2.32%	1.20±0.45%	2.34±2.20%

Consistent with the appearance of the overlaid spectra, the average linewidths with coefficients of variation of 10-20% indicate good spectroscopic quality, consistent shimming and good reproducibility. The larger % CRLB for the Nottingham data are a consequence of using a more complex basis set with more components than were used for the jMRUI fitting for the Manchester and Cardiff data. There is a decrease in Bias and increase in Variance as more components are added into the basis set.

NAA SNR is reported for the short-echo ACC spectra in Table S8, below. Here the data are reported by site and by diagnostic group. Interpretation of the clear SNR increase at 7T compared to 3T is not straightforward: STEAM (used at 7T) has half the signal of PRESS (3T), but the echo-time is shorter

(17ms compared to 35ms) and more scans were averaged at 7T (288 compared to 128), but at a wider bandwidth (4kHz compared to 2kHz, which reduces SNR by about 30%). It is also likely that the receiver coils at 3T are more efficient having had more development history, nevertheless it is somewhat surprising that the SNR improvement at 7T is relatively modest. It is tempting to suggest there is an age effect on SNR (higher in younger cohorts), but investigation of this is beyond the scope of this work.

Table S8 NAA SNR in ACC spectra

Site/Group	Early Onset	Established disease	Young Healthy Controls	Old Healthy controls
Cardiff	284±49	232±60	256±46	265±87
Manchester	318±87	247±50	348±68	251±68
Nottingham	351±51	342±122	411±78	323±50

The fitted linewidths and % CRLB are reported below (Table S9) for GABA and glutathione measured using MEGAPRESS in Cardiff and Manchester.

Table S9 Quality metrics for MEGAPRESS spectra.

Metabolite / Site	GABA		Glutathione	
	LW (Hz)	% CRLB	LW (Hz)	% CRLB
Cardiff	16.1±1.4	4.3±3.1	13.7±1.8	5.5±3.2
Manchester	20.3±2.3	4.8±1.3	9.7±2.4	14.4±6.4

The linewidth of the edited GABA signal is significantly larger than the N-acetylaspartate and water linewidths recorded in the same sessions. This is because the GABA signal includes a contribution from co-edited macromolecules (commonly referred to as GABA+) which was fit as a single Gaussian line. As expected from the signal-to-noise ratios of the two acquisitions and the greater complexity of the glutathione spectrum, GABA is fitted with better precision than glutathione.

Supplementary References

1. Lin A, Andronesi O, Bogner W, Choi IY, Coello E, Cudalbu C, et al. Minimum Reporting Standards for in vivo Magnetic Resonance Spectroscopy (MRSinMRS): Experts' consensus recommendations. NMR Biomed. 2021;34(5):e4484.
2. Bottomley PA. Spatial localization in NMR spectroscopy in vivo. Ann N Y Acad Sci. 1987;508:333-48.
3. Mescher M, Merkle H, Kirsch J, Garwood M, Gruetter R. Simultaneous in vivo spectral editing and water suppression. NMR Biomed. 1998;11(6):266-72.
4. Mullins PG, McGonigle DJ, O'Gorman RL, Puts NA, Vidyasagar R, Evans CJ, et al. Current practice in the use of MEGA-PRESS spectroscopy for the detection of GABA. Neuroimage. 2014;86:43-52.

5. Terpstra M, Henry PG, Gruetter R. Measurement of reduced glutathione (GSH) in human brain using LCModel analysis of difference-edited spectra. Magnetic resonance in medicine. 2003;50(1):19-23.
6. Bruhn H, Frahm J, Gyngell ML, Merboldt KD, Hanicke W, Sauter R. Localized proton NMR spectroscopy using stimulated echoes: applications to human skeletal muscle in vivo. Magnetic resonance in medicine. 1991;17(1):82-94.
7. Brant-Zawadzki M, Gillan GD, Nitz WR. MP RAGE: a three-dimensional, T1-weighted, gradient-echo sequence--initial experience in the brain. Radiology. 1992;182(3):769-75.
8. Edden RA, Puts NA, Harris AD, Barker PB, Evans CJ. Gannet: A batch-processing tool for the quantitative analysis of gamma-aminobutyric acid-edited MR spectroscopy spectra. J Magn Reson Imaging. 2014;40(6):1445-52.
9. Harris AD, Puts NA, Edden RA. Tissue correction for GABA-edited MRS: Considerations of voxel composition, tissue segmentation, and tissue relaxations. J Magn Reson Imaging. 2015;42(5):1431-40.
10. Mierisova S, van den Boogaart A, Tkac I, Van Hecke P, Vanhamme L, Liptaj T. New approach for quantification of short echo time in vivo 1H MR spectra of brain using AMARES. NMR Biomed. 1998;11:32-9.
11. Ratiney H, Sdika M, Coenradie Y, Cavassila S, van Ormondt D, Graveron-Demilly D. Time-domain semi-parametric estimation based on a metabolite basis set. NMR in biomedicine. 2005;18(1):1-13.
12. Starcukova J, Stefan D, Graveron-Demilly D. Quantification of short echo time MRS signals with improved version of QUantitation based on quantum ESTimation algorithm. NMR in biomedicine. 2023;36(11):e5008.
13. Stefan D, Di Cesare F, Andrasescu A, Popa E, Lazariev A, Vescovo E, et al. Quantitation of magnetic resonance spectroscopy signals: the jMRUI software package

Measurement Science and Technology

2009;20(10):104035–44.
14. Provencher SW. Estimation of metabolite concentrations from localized in vivo proton NMR spectra. Magnetic resonance in medicine. 1993;30(6):672-9.
15. Mikkelsen M, Singh KD, Brealy JA, Linden DE, Evans CJ. Quantification of gamma-aminobutyric acid (GABA) in (1) H MRS volumes composed heterogeneously of grey and white matter. NMR in biomedicine. 2016;29(11):1644-55.
16. Sanaei Nezhad F, Anton A, Parkes LM, Deakin B, Williams SR. Quantification of glutathione in the human brain by MR spectroscopy at 3 Tesla: Comparison of PRESS and MEGA-PRESS. Magnetic resonance in medicine. 2017;78(4):1257-66.
17. Kumar J, Liddle EB, Fernandes CC, Palaniyappan L, Hall EL, Robson SE, et al. Glutathione and glutamate in schizophrenia: a 7T MRS study. Mol Psychiatry. 2020;25(4):873-82.
18. Ashburner J, Friston KJ. Unified segmentation. Neuroimage. 2005;26(3):839-51.
19. Gasparovic C, Song T, Devier D, Bockholt HJ, Caprihan A, Mullins PG, et al. Use of tissue water as a concentration reference for proton spectroscopic imaging. Magn Reson Med. 2006;55(6):1219-26.
20. Kaiser LG, Marjanska M, Matson GB, Iltis I, Bush SD, Soher BJ, et al. (1)H MRS detection of glycine residue of reduced glutathione in vivo. J Magn Reson. 2010;202(2):259-66.
